# An MXene-Grafted Terpolymer Hydrogel for Adsorptive Immobilization of Toxic Pb(II) and Post-Adsorption Application of Metal Ion Hydrogel

**DOI:** 10.3390/gels9100827

**Published:** 2023-10-19

**Authors:** Himarati Mondal, Mrinmoy Karmakar, Bhaskar Datta

**Affiliations:** 1 Department of Chemistry, Indian Institute of Technology Gandhinagar, Palaj 382055, Gujarat, India; 2 Department of Chemical Engineering, Indian Institute of Technology Gandhinagar, Palaj 382055, Gujarat, India; 3 Presently in Department of Pharmacy, College of Pharmacy, Kangwon National University, Chuncheon 24341, Gangwon, Republic of Korea; 4 Department of Biological Engineering, Indian Institute of Technology Gandhinagar, Palaj 382055, Gujarat, India

**Keywords:** composite hydrogel, Pb(II) adsorption, wastewater treatment, electrode material, adsorption isotherm

## Abstract

Toxic metal ions present in industrial waste, such as Pb(II), introduce deleterious effects on the environment. Though the adsorptive removal of Pb(II) is widely reported, there is a dearth of research on the suitable utilization and disposal of the Pb(II)-adsorbed adsorbent. In this work, an MXene-grafted terpolymer (**MXTP**) hydrogel has been designed for the adsorption of Pb(II) under ambient conditions of pH and temperature. The hydrogel **MXTP** was synthesized by facile one-pot polymerization in aqueous solvent, and the detailed structural characterization of terpolymer (**TP**), **MXTP**, and Pb(II)-loaded **MXTP**, i.e., Pb(II)-**MXTP**, was carried out by a combination of proton nuclear magnetic resonance (^1^H NMR), Fourier-transform infrared (FTIR), X-ray photoelectron spectroscopy (XPS), X-ray diffractometric (XRD), thermogravimetric/differential thermogravimetric (TG/ DTG), and field emission scanning electron microscopic (FESEM) analyses. The specific capacitance and conductivities of Pb(II)-**MXTP** were studied with cyclic voltammetry (CV) and electrical impedance spectroscopy (EIS), which unambiguously indicate successful post-adsorption application. The specific capacitance of **MXTP** decreased after Pb(II) adsorption, whereas the conductivity increased significantly after Pb(II) adsorption, showing that **MXTP** can be successfully deployed as a solid electrolyte/anode after Pb(II) adsorption. This study covers the synthesis of a novel MXene-grafted terpolymer hydrogel for adsorptive exclusion of Pb(II) and assessment of the as-adsorbed Pb(II)-loaded hydrogel as a solid electrolyte/anode material and is the first demonstration of such post-adsorptive application.

## 1. Introduction

Dissolved metal ions in effluents of foundation industries, such as mining, metallurgy, steel, coal-based power, glass, electroplating and dyes and pigments carry severe environmental risks, especially when released into natural water resources without suitable treatment [1,2]. Lead (Pb) is considered the second most toxic metal and is highly noxious and non-disintegrative while comprising only 0.002% of the Earth’s crust [3,4]. Pb has no known biological function and is hazardous for plants, animals, and human beings. Pb is toxic to plants beyond 30 mg/kg of soil concentration [4]. In plants, Pb strongly inhibits seed germination and development, root elongation, plant growth, transpiration, chlorophyll production, and water/protein content [3,4,5]. In human beings, Pb exerts detrimental effects on the brain, kidney, liver, and central nervous system, along with anemia, dizziness, headache, irritability, and infertility [6]. Therefore, research for removing Pb(II) via easy-to-use, effective, and sustainable technologies is of immediate environmental, societal and public health relevance. The commonly adopted techniques for Pb(II) removal include reverse osmosis, reduction, solvent extraction, coagulation, ion exchange, chemical precipitation, membrane-based separation, and adsorption [7,8]. Of these, adsorption is extensively accepted as an efficient, cost-effective and easy-to-deploy method, whose operational cost can be controlled via the reusability of adsorbents [9,10,11]. Hydrogels are a distinctive class of polymeric materials that exhibit variegated swelling and can be engineered with chemical functionalities to facilitate the adsorption of specific analytes [12]. Accordingly, hydrogels are capable of detoxifying industrial effluents [13]. Poly[acrylic acid], i.e., PAA, is a low-cost biocompatible super-adsorbing pH-responsive anionic polymer, extensively studied for preparing pH-sensitive hydrogels [14,15,16,17,18]. While the presence of easily ionizable -COOH functionalities of PAA make them pH-responsive, the higher water solubility, poor temperature responsiveness, and non-tunable mechanical properties limit their direct employability. Again, poly[2-hydroxyethyl methacrylate] (PHEMA)-based hydrogels are biocompatible, stable and possess high swelling abilities and tunable mechanical properties [19]. The pendant O–H functionalities of PHEMA hydrogels serve as suitable sites for the adsorption of polar adsorbates, such as metal ions [20]. Therefore, the copolymer of AA and HEMA, i.e., poly[AA-co-HEMA], should possess tunable mechanical properties accompanied by high pH responsiveness and adsorption capacity (AC) attributed to –COOH and O–H functionalities. Nevertheless, the high population of hydrophilic moieties in poly[AA-co-HEMA] results in appreciable water solubility constraining the repetitive adsorption–desorption during adsorptive recovery of metal ions including Pb(II) from industrial wastewater. Thus, while poly[AA-co-HEMA] may exhibit superior sustainability in dehydrated state, its sustainability of use in contact with aqueous solutions of pollutants is questionable. This challenge has triggered the need to synthesize a polymer composite with a reinforcing agent. Two-dimensional materials have shown promise in this regard based on the sustainability of use, mechanical properties, electrical conductivity, and specific capacitance [21].

Among different 2D materials, the family of transition metal carbides commonly known as MXenes are recently discovered materials possessing tunable metallic conductivity, solution processability, structural robustness, and aspect ratios [22,23,24,25]. The general chemical formula of MXene is M_n+1_X_n_T_x_ (*n* = 1–4), where M, X, and T represent early transition metals (such as Zr, Ti, V, Ta, Nb, and Mo), carbon and/or nitrogen, and pendent termination functional groups (such as O–H and F), respectively [26,27,28]. Among the discovered MXenes, Ti_3_C_2_T_x_ is mostly studied to date [29]. The two-dimensional MXene is generally synthesized by exfoliating the ‘‘A’’ layer from MAX-phase via HF-aided etching reaction at high temperatures. In MAX, the letter “A” stands for the interlaying Al^3+^ layers, which are eliminated during etching [30]. MAX phases can be denoted as alternately stacked ‘‘MX’’ layers and close-packed ‘‘A’’ atomic layers, represented by the general formula of M_n+1_AX_n_, where M–X (ionic/covalent) bonds are more stable than those of M–A (metallic) bonds [16]. MXene-incorporated polymeric hydrogels have been reported with versatile properties including photo-redox catalysis, photothermal behavior, and sensing [26]. For instance, MXene/poly[AM-co-LMA]/PNIPA hydrogel, a nanomaterial containing a double network hydrogel, has been reported to exhibit attractive conductivity and capacitance [30]. Again, MXene/poly[AM-co-PVA] hydrogel has been used to synthesize antifreeze material, which can be stored at −40 °C [31]. An MXene/PAM hydrogel has been studied for the delivery of antimicrobial chloramphenicol drugs [32].

In this work, we have used an MXene-grafted hydrogel for the adsorption and reuse of Pb(II). Compared to adsorption itself, the post-adsorptive use of Pb(II) is not as widely reported. Desorption of the Pb(II) followed by recycling or reuse of the adsorbent is reported albeit for 5–6 cycles. The subsequent disposal of the adsorbent in landfills, often bearing non-trivial amounts of Pb(II), is fraught with risks. To solve this problem, we have tested a Pb(II)-adsorbed MXene-grafted hydrogel as a prospective solid electrolyte/anode material. The design of our MXene-grafted poly[AA-co-HEMA], i.e., **MXTP**, is based on the need to introduce conducting properties in the resulting adsorbent. The specific capacitance and conductivity of Pb(II)-**MXTP** highlight the hitherto unreported class of MXene-grafted hydrogels as promising Pb(II) adsorbents for sustainable post-adsorptive applications.

## 2. Results and Discussion

### 2.1. NMR Analysis

**Figure 1:**
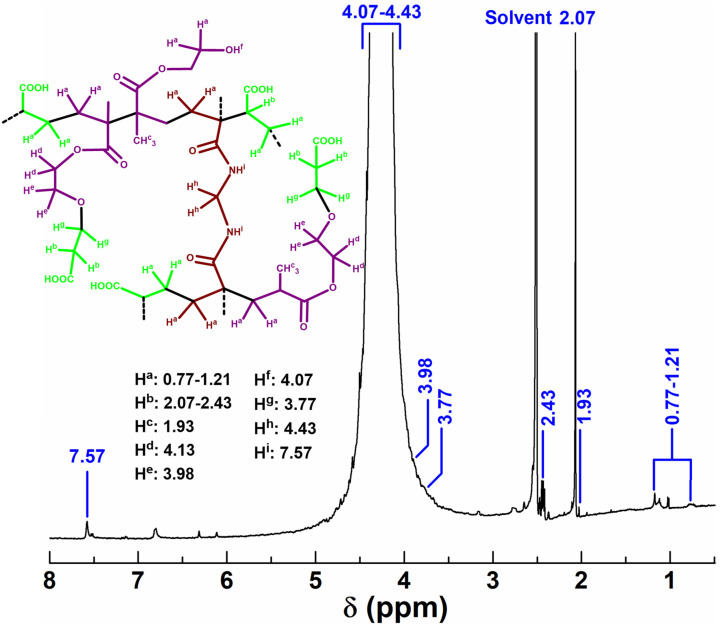
^1^H NMR of **TP** performed in DMSO-d_6_ solvent at 500 MHz.

In **TP**, the formation of –H_2_C(β)–CH_2_(α)–CO– and –H_2_C(β)–CH(CO)– moieties from H_2_C=CH–CO– via C–C coupled polymerization was inferred from (–C**H**_2_(α)–/>C**H**–) and –C**H**_2_(β)– peaks within 2.07–2.43 and 0.77–1.21 ppm (Figure 1), respectively, as well as the obsolescence of vinyl-proton-specific peaks within 6.03–6.59, 5.53–6.15, and 5.59–6.13 ppm for AA, HEMA, and MBA, respectively [10,33]. Again, in **TP**, peaks at 4.13/3.98 and 1.93 ppm attributed to –C**H**_2_– and –C**H**_3_ of HEMA confirmed the inclusion of HEMA in **TP**. Similarly, the presence of MBA in TP was inferred from the characteristic –C**H**_2_– peak of MBA at 4.43 ppm. Importantly, the O–C coupled in situ attachment of the third comonomer, i.e., (3-(2-((2-methylbutanoyl)oxy)ethoxy)propanoic acid)/MBOEPA, could be inferred from –CH_2_CH(CH_3_)COOCH_2_CH_2_OC**H**_2_CH_2_COOH peak at 3.77 ppm in **TP** [19,20]. Moreover, the characteristic N–**H** and O–**H** peaks of MBA and HEMA occurred at 7.57 and 4.07 ppm in **TP**, respectively.

### 2.2. FTIR Analysis

**Figure 2:**
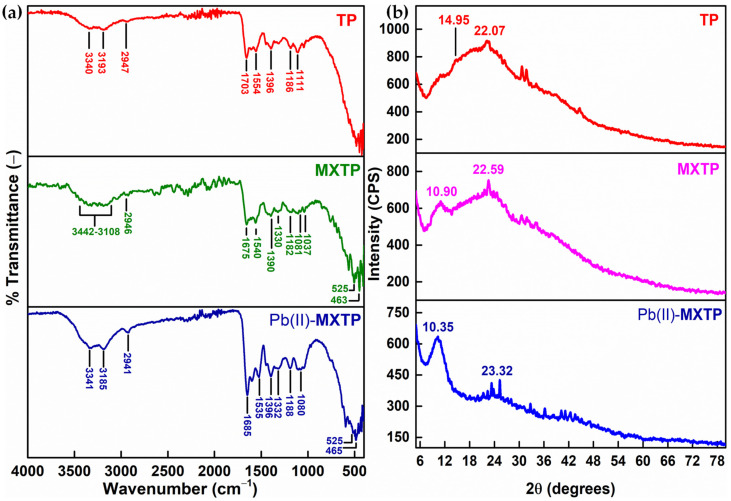
(**a**) FTIR and (**b**) XRD of **TP**/ **MXTP**/ Pb(II)-**MXTP**.

In FTIR of MAX (Figure S1 and Table 1), peaks at 497/420 and 463 cm^−1^ could be related to Al–C and Ti–C bonds, respectively [34]. Disappearance of Al–C bond-specific peaks after etching and simultaneous arrival of new peaks at 3455, 1350, and 528 cm^−1^, attributed to O–H *str.*, C–F band, and Ti–O band, respectively, envisaged the elimination of Al^3+^ planes from MAX and formation of O–H and F^−^ terminal functionalities in MXene [35,36,37,38]. The presence of AA and HEMA in **TP** and **MXTP** could be justified from hydrogen-bonded >C=O *str.* of –COOH, >C=O *asym. str.* of –COO^−^, and –CH_2_– *def.* band of –CH_2_–CO–O– group of ester at 1703, 1554, and 1396 cm^−1^ in **TP** and 1675, 1540, and 1390 cm^−1^ (Figure 2a) in **MXTP**, respectively [36]. The shifting in >C=O *asym. str.* of –COO^−^ of **TP** after MXene incorporation could be related to the possible coordination of –COO^−^ with Ti(IV) of MXene. The terminal O–H functionalities of MXene provided additional hydrogen bonding possibilities and hence stronger hydrogen bonding could be envisaged through a broader O–H *str.* peak in **MXTP**. The O–C coupled incorporation of the third comonomer, i.e., MBOEPA, was envisaged from C–O–C *asym. str.* and *sym. str.* peaks at 1186/1182 and 1111/1081 cm^−1^ in **TP**/**MXTP**, respectively [19,36]. Such an observation was observed earlier from ^1^H NMR analysis, in which the peak of –CH_2_CH(CH_3_)COOCH_2_CH_2_OC**H**_2_CH_2_COOH at 3.77 ppm in **TP** denoted the in situ O–C coupled incorporation of MBOEPA. During Pb(II) adsorption at pH > pH_PZC_ (pH_PZC_ = 5.93, Figure S2), considerable deprotonation of –COOH of AA and O–H of MXene occurred, resulting in a significant reduction in hydrogen bonding. Two distinct O–H *str.* peaks at 3341 and 3185 cm^−1^ were observed in Pb(II)-**MXTP** attributed to the O–H *str.* of terminal O–H and –COOH, respectively [35,39]. The shifting of >C=O *asym. str.* of –COO^−^ could be related to the coordination of –COO^−^ with Pb(II) through various modes.

### 2.3. XRD Analysis

In the XRD profile of MAX, peaks at 2θ = 9.67, 19.28, 29.69, 34.22, 36.91, 38.93, 41.91, 48.77, 56.59, and 60.37° (Figure S3) closely resembled the standard JCPDS file No. 52-0875 suggesting the prevalent hexagonal MAX phases [41]. After etching with (CaF_2_ + HCl), the obsolescence of the peak at 2θ = 38.93° (Figure S3) in MXene indicated the removal of Al^3+^-planes from MAX [41], observed earlier from the vanishing of Al–C specific FTIR peaks at 497/420 cm^−1^, respectively. Again, peaks at 2θ = 18.17, 27.37 and 61.12° in MXene represented the crystalline planes of Ti_3_C_2_ [41]. Notably, the broadening of XRD peaks in MXene compared to those of MAX indicates the prevalence of exfoliation.

In the XRD profile of **TP**, peaks at 2θ = 14.95 and 22.07° (Figure 2b) were attributed to properly organized polymer planes by virtue of hydrogen bonding. Notably, the arrival of the sharp MXene-specific peak at 2θ = 10.90° reflected the introduction of MXene within the matrix of **MXTP**. The hydrogen-bonded orderly arrangement of polymer chains could also be observed in **MXTP**. Notably, the prevalence of hydrogen bonding in **TP** and **MXTP** was already described in FTIR analysis. After Pb(II) adsorption, the change in the environment of –COOH and –OH resulted in the shifting of the peak from 2θ = 22.59 of **MXTP** to 23.32° in Pb(II)-**MXTP**. Surprisingly, the absence of Pb(II)-specific sharp peaks in the XRD spectrum of Pb(II)-**MXTP** indicated the superficial depositions of non-crystalline Pb(II) complexes.

### 2.4. XPS Analysis

**Figure 3:**
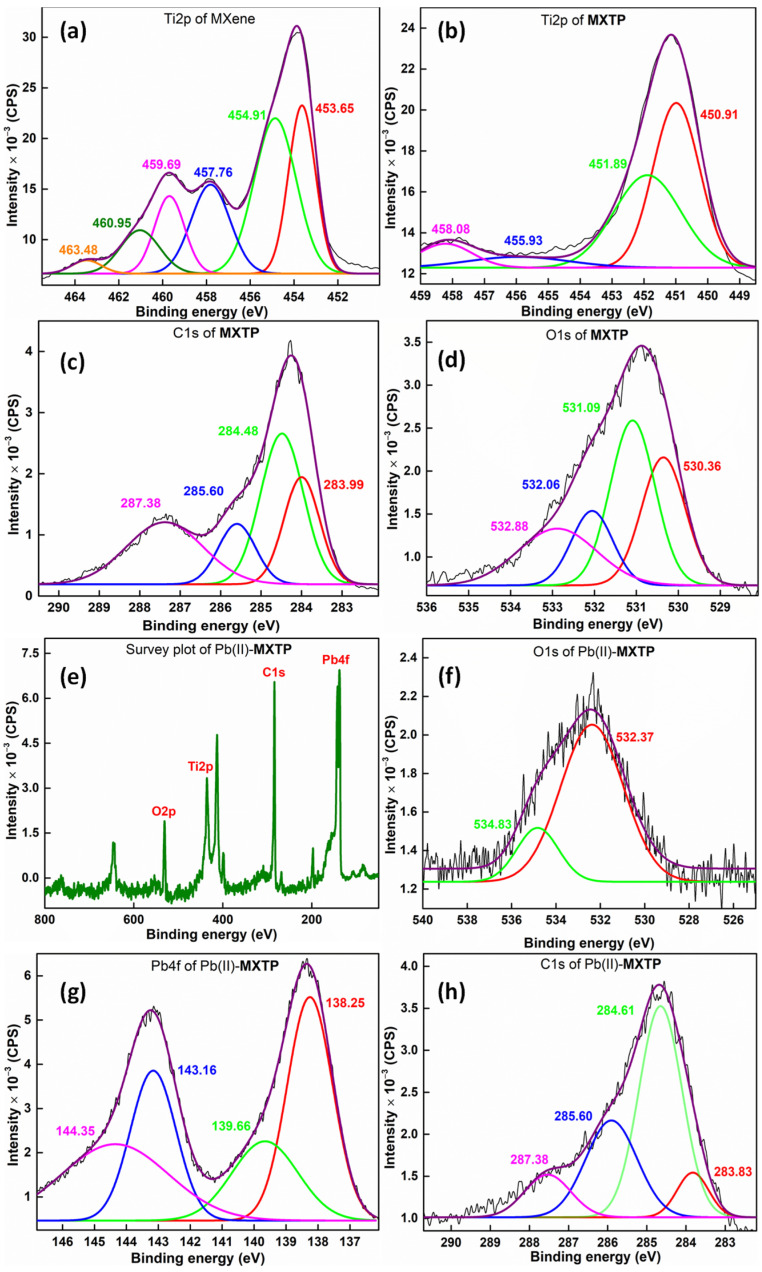
XPS of (**a**/**b**) Ti2p of MXene/**MXTP**, (**c**/**h**) C1s of **MXTP**/Pb(II)-**MXTP**, (**d**/**f**) O1s of **MXTP**/Pb(II)-**MXTP**, and (**g**) Pb4f of Pb(II)-**MXTP** and (**e**) survey plot.

XPS was carried out with the primary objective of confirming the presence of MXene in **MXTP**. The binding pattern of MXene with **TP** was identified by observing changes in Ti2p BEs in MXene and **MXTP**. The deconvoluted Ti2p of MXene contained three sets of Ti2p_3/2_/Ti2p_1/2_ peaks at BE = 453.65/459.69, 454.91/460.95, and 457.76/463.48 eV (Figure 3a, Table 2), attributed to **Ti**–C–T_o_, **Ti**–C–T_o,F_, and **Ti**–C–T_F_, respectively (**Ti**–C–T_o_, **Ti**–C–T_F_, and **Ti**–C–T_o,F_ indicate terminal O–H, F^−^, and O–H + F^−^, respectively) [42]. The spin–orbit coupling of Ti2p_3/2_ and Ti2p_1/2_ peaks varied within 5.72–6.04 eV. The presence of O–H and F^−^ terminal groups in MXene could be envisaged from this fitting pattern, which was also deduced earlier from FTIR analysis. In **MXTP**, the presence of Ti could be inferred from the appearance of a Ti-specific peak at BE ~450 eV in the wide-scan survey plot of **MXTP** (Figure S4). Importantly, no such peak is present in the wide-scan survey plot of **TP** (Figure S4), which also supported that the peak ~450 eV in the survey plot of **MXTP** originated from Ti. After internalization within the matrix of **TP**, the Ti2p_3/2_/Ti2p_1/2_ peaks shifted to 450.91/455.93 and 451.89/458.08 eV (Figure 3b), respectively [42]. The shifting of Ti2p peaks towards the lower BEs indicated the coordinative attachment of Ti(IV) with **TP** during **MXTP** formation.

In **MXTP**, the deconvoluted C1s spectrum was composed of four peaks at BE = 283.99, 284.48, 285.60, and 287.38 eV (Figure 3c) because of metal **carbide** [43], –**C**H_2_–/–**C**H< [40,44], –**C**–O–**C** [11,20], and –**C**OOH/–**C**OO^−^ [6,40,45,46], respectively. Of these, the metal carbide peak indicated incorporation of MXene in **MXTP**, whereas the BE for the **C**–O–**C** moiety substantiated the in situ O–C coupled formation of the new comonomer, i.e., MBOEPA, inferred earlier from –CH_2_CH(CH_3_)COOCH_2_CH_2_OC**H**_2_CH_2_COOH peak at 3.77 ppm of ^1^H NMR and C–O–C *asym.*/*sym. str.* peaks at 1182/1081 cm^−1^ of FTIR analyses. The deconvoluted O1s spectrum of **MXTP** possessed four peaks at BEs = 530.36, 531.09, 532.06, and 532.88 eV (Figure 3d), attributed to –C(=**O**)–OH [9], –C**OO**^−^ [11,45], **O**–H termination group of MXene [47], and –C(=O)–**O**H [11,48,49], respectively.

The adsorption of Pb(II) could be justified by the presence of a Pb-specific peak at ~140 eV in the wide-scan survey plot of Pb(II)-**MXTP** (Figure 3e). No peak was present in the survey plots of **TP** and **MXTP** in this range (Figure S4). In fact, XPS played an important role in justifying the bonding pattern of Pb(II) during its adsorption onto **MXTP**. After Pb(II) adsorption at pH > pH_PZC_, the O1s BE of –C**OO**^−^ increased significantly from 531.09 to 532.37 eV (Figure 3f) indicating the existence of coordinate bonding between –C**OO**^−^ of **MXTP** and Pb(II) during adsorption, observed earlier from the shifting of >C=O *asym. str.* of –COO^−^ from 1540 cm^−1^ of **MXTP** to 1535 cm^−1^ in Pb(II)-**MXTP**. This phenomenon was further supported by the decrease in Pb4f BEs of Pb4f_5/2_ and Pb4f_7/2_ from 143.80 and 139.30 eV of Pb(NO_3_)_2_ to 143.16 and 138.25 eV, respectively (Figure 3g), in Pb(II)-**MXTP** [1,6]. Again, Pb4f_5/2_ and Pb4f_7/2_ BEs at 144.35 and 139.66 eV in Pb(II)-**MXTP** envisaged the weaker interaction between Pb(II) and **MXTP** via the ion exchange mechanism [1].

### 2.5. TGA Explanation

**Figure 4:**
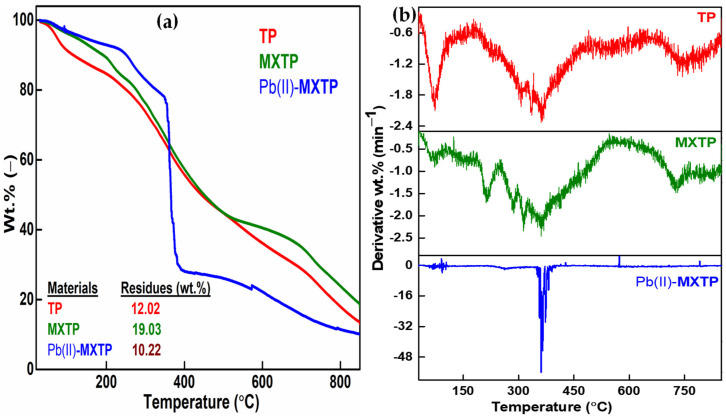
(**a**) TGA and (**b**) DTG of **TP**/**MXTP**/Pb(II)-**MXTP**.

From TG analysis, the faster rate of thermal degradation of **TP** than that of **MXTP** up to 120 °C (Figure 4a) could be related to the weaker hydrogen bonding in **TP**, observed earlier from FTIR and XRD analyses. Such an observation could be further justified by DTG peaks around 74 °C having degradation rates of 2.13 and 0.87 wt.% min^−1^ for **TP** and **MXTP**, respectively (Figure 4b). The presence of terminal O–H and F^−^ functionalities in **MXTP** resulted in such enhanced hydrogen bonding. In the next major degradation range within 250–483 °C, the weight loss was associated with the evaporation of H_2_O and CO_2_ because of the formation of acid into anhydride and its subsequent decomposition, respectively [50]. In this degradation zone, the improved thermostability of **MXTP** compared to **TP** might be related to coordinate bonding between Ti(IV) and –COO^−^ of **TP**. The enhanced thermostability of **MXTP** compared to that of **TP** beyond 517 °C indicated the reinforcement of the polymer backbone by MXene. Finally, the presence of thermostable inorganic Ti(IV) ions of MXene in **MXTP** resulted in a significantly higher residue amount (19.03 wt.%) compared to 12.02 wt.% of **TP**.

After adsorption of Pb(II) at pH > pH_PZC_, the significant reduction in hydrogen bonding as reported from FTIR analysis would be expected to intensify the removal rate of vapor, resulting in the more rapid thermal degradation of Pb(II)-**MXTP** compared to **MXTP**. This expected behavior could be confirmed by the degradation rate of 4.69 wt.% min^−1^ of Pb(II)-**MXTP** compared to 2.13 wt.% min^−1^ of **MXTP** (Figure 4b). However, almost similar thermal stability was observed for Pb(II)-**MXTP** and **MXTP** up to 111 °C. Importantly, the improved thermostability of Pb(II)-**MXTP** compared to that of **MXTP** within 111–349 °C can be related to the formation of a Pb(II)-acetate type complex in Pb(II)-**MXTP**, which restricts acid to anhydride formation and its decomposition [1,2]. Formation of coordinate bonding within Pb(II) and –COO^−^ during adsorption has already been established from shifting of O1s of –C**OO**^−^, Pb4f_7/2_, and Pb4f_5/2_ Bes from 531.09, 143.80, and 139.30 eV of **MXTP** to 532.37, 143.28, and 138.49 eV in Pb(II)- **MXTP** (Figure 3) in XPS as well as from shifting of >C=O *asym. Str.* Of –COO^−^ from 1540 cm^−1^ of **MXTP** to 1535 cm^−1^ in Pb(II)-**MXTP** in FTIR. Moreover, the Pb(II)-acetate type complex contained crystal water, which could not be eliminated within 120 °C [51]. Therefore, the expected initial poor thermostability originating from the lack of hydrogen bonding was made up by the retention of crystal water within the Pb(II)-acetate type complex in Pb(II)-**MXTP**. For **MXTP**, three distinct DTG peaks at 212, 286, and 314 °C were observed as having rates of mass loss of 1.74, 1.92, and 2.32 wt.% min^−1^, respectively, within 111–349 °C (Figure 4b). However, the mere DTG peak at 262 °C with the rate of mass loss of 2.04 wt.% min^−1^ in Pb(II)-**MXTP** justified the elevated thermostability of Pb(II)-**MXTP** compared to that of **MXTP** within this temperature range. Nevertheless, the sharp weight loss (47.02 wt.%) within 350–388 °C and the corresponding sharp DTG peak at 361 °C (rate of mass loss = 56.14 wt.% min^−1^) in Pb(II)-**MXTP** indicated the degradation of Pb(II)-acetate [52]. In fact, the steady decomposition of Pb(II)-**MXTP** up to 800 °C was attributed to the multistage decomposition of Pb(II)-acetate to generate PbO [51]. 

### 2.6. FESEM Assessment

**Figure 5:**
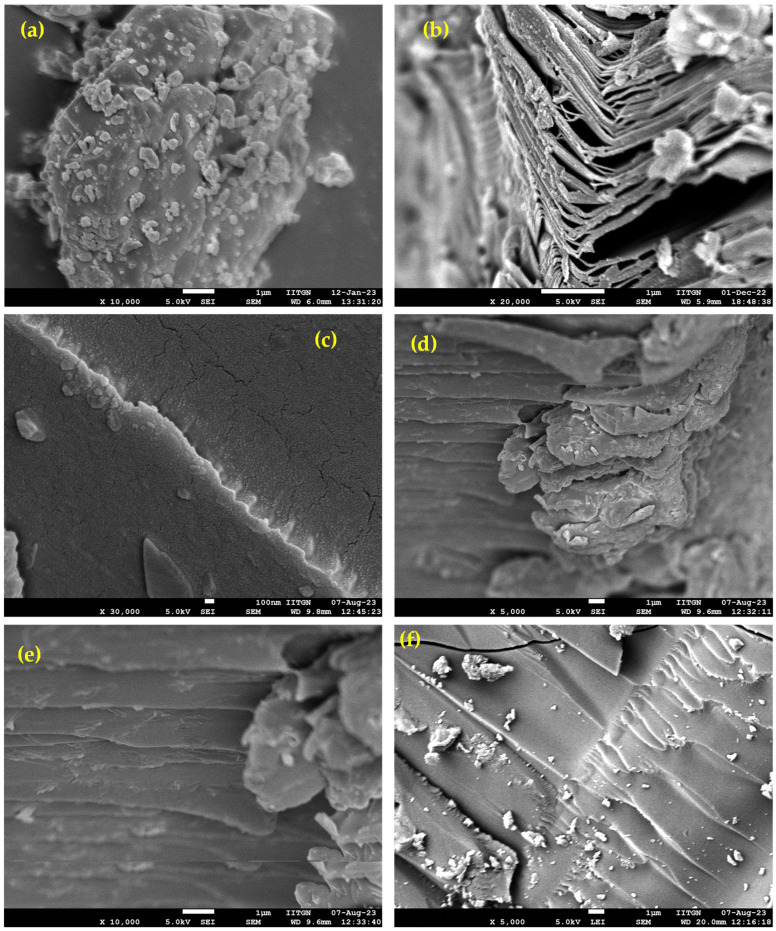
FESEM photomicrographs of (**a**) MAX, (**b**) MXene, (**c**) **TP**, (**d**,**e**) **MXTP** (lower/higher magnifications), and (**f**) Pb(II)-**MXTP**.

The etching of MAX (Figure 5a) by CaF_2_ + HCl resulted in the formation of tightly bound parallel planes, which are clearly visible in the FESEM photomicrograph of MXene (Figure 5b). Distinct alternative planes of Ti(IV) and carbides were generated via etching with in situ generated HF. In **TP**, featureless dense morphology without the distinct phase boundary could be visualized (Figure 5c), because of the C–C/O–C coupled covalent attachment of monomers. However, in **MXTP** (Figure 5d), the characteristic comb-like structure was visualized, suggesting the internalization of MXene with **TP**. The protruded plane-like appearance of MXene could be observed in the zoomed morphology of **MXTP** (Figure 5e). The superficial deposition of irregularly shaped Pb(II)-acetate complexes could be visualized in the photomicrograph Pb(II)-**MXTP** (Figure 5f). The existence of such irregular/non-crystalline shapes of Pb(II)-acetate complexes might be the reason for the absence of sharp peaks in the XRD of Pb(II)-**MXTP**.

### 2.7. Adsorption of Pb(II) by MXTP

**Figure 6:**
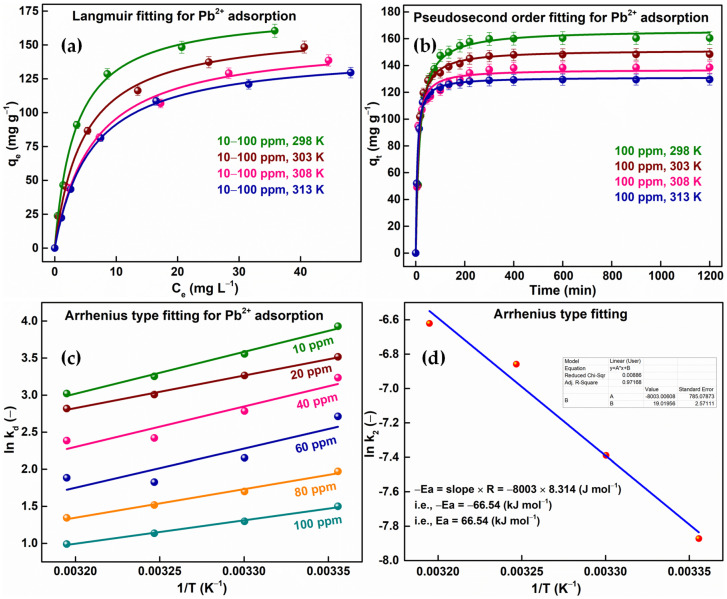
Statistical analyses of adsorption of Pb(II) onto **MXTP**: (**a**) isotherm (Langmuir model), (**b**) kinetics (pseudo-second model), and Arrhenius type fitting for evaluating (**c**) thermodynamic parameters and (**d**) activation energy (*E_a_*).

The equilibrium adsorption data were fitted to different isotherm models, such as Langmuir, Henry, Freundlich, Sips, and BET [1,42], of which the Langmuir model displayed the best fit due to the highest adjusted *R*^2^/*F* and lowest *χ*^2^ values [6,9]. The maximum AC, i.e., *q_max_*, varied as 175.36, 162.44, 154.21, and 145.41 mg g^−1^ at 298, 303, 308, and 313 K (Figure 6a and Table 3), respectively. Since the Langmuir model depends on the presumption of monolayer adsorption only, the adsorption of Pb(II) onto the surface of **MXTP** was also assumed to be monolayered [1,2]. Again, the better fitting of kinetics data with the pseudo-second-order kinetics model compared to fitting with the pseudo-first-order kinetics model (Figure 6b), and attainment of the activation energy of 66.54 kJ mol^−1^ (Figure 6d), indicated chemical bonding behind the adsorption of Pb(II) by **MXTP**. Pseudo-second-order rate constants, i.e., *k*_2_, followed an increasing trend with the increase in temperature because of the faster adsorption at elevated temperatures. The exothermic and spontaneous nature of chemisorption is justified by the negative values of Δ*H*^0^ and Δ*G*^0^ at the entire working temperature (Figure 6c and Table 4).

### 2.8. Comparison of the Results

A brief literature study for the adsorption of Pb(II) by using various low-cost polymer materials, such as nanoparticles, clays, zeolites, chemically modified inorganic materials, and agricultural wastes, at varying initial concentrations (i.e., 5–1000), temperatures (i.e., 295–303 K), and pH_o_ (i.e., 4.0–7.0) is detailed in Table 5. From this study, the maximum adsorption capacity of **MXTP** can be concluded as the highest among those of the previously reported adsorbents.

### 2.9. Desorption of Pb(II) from Pb(II)-MXTP and Reusability Studies of MXTP

The primary driving force of the adsorption of Pb(II) by **MXTP** is coordinate bonding between –COO^−^/–O^−^ of **MXTP** and Pb(II). Therefore, controlling the pH of the solution should be the key factor in desorbing the already adsorbed Pb(II). Actually, at pH < pH_PZC_, the electrostatic repulsion between positively charged **MXTP** and adsorbed Pb(II) would cause the detachment of Pb(II). Therefore, desorption was tried at pH 2, leading to ~95% desorption. Thereafter, **MXTP** was applied further for adsorption at pH 7, and again, desorption was tried at pH 2. Such iterative adsorption–desorption experiments were conducted to confirm the reusability of **MXTP**. Remarkably, **MXTP** achieved more than 92% adsorption even after the fifth adsorption–desorption cycle.

### 2.10. Capacitance Studies from CV Experiments

**Figure 7:**
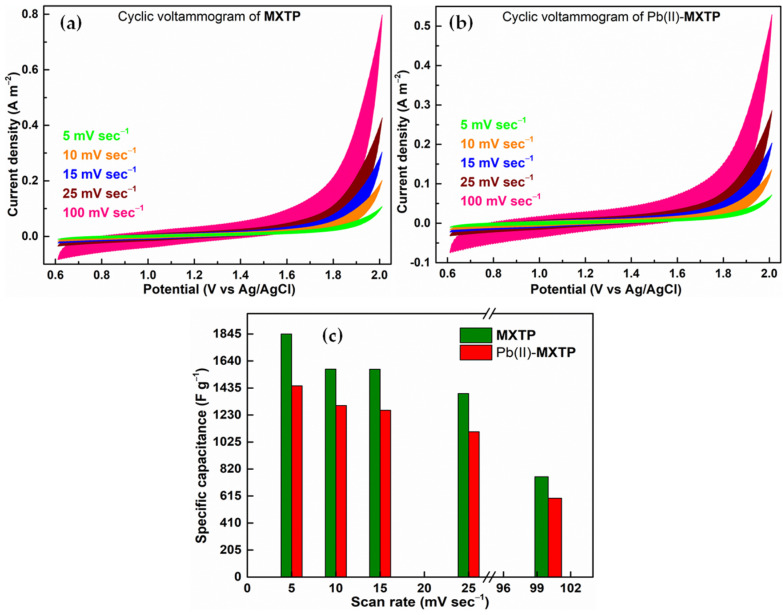
(**a**,**b**) CV plots of **MXTP**/Pb(II)-**MXTP** and (**c**) variation in specific capacitances of **MXTP**/Pb(II)-**MXTP**.

CV experiments of **MXTP** and Pb(II)-**MXTP** were carried out to study the effect of adsorbed Pb(II) on the specific capacitance of **MXTP**. From cyclic voltammograms of both **MXTP** and Pb(II)-**MXTP** (Figure 7a,b), current densities were found to increase with the increase in scanning rates from 5 to 100 mV sec^−1^. However, since *C_p_* is inversely proportional to scanning rates (Text S1), *C_p_* values were found to decrease significantly with the increasing scanning rates from 5 to 100 mV sec^−1^. The *C_p_* values of **MXTP** were found to be 1844.65, 1579.02, 1577.87, 1392.79, and 760.88 F g^−1^ at 5, 10, 15, 25, 100 mV sec^−1^, respectively, which are significantly higher than the other MXene-based polymers. Therefore, the as-synthesized MXene-grafted polymer system can be successfully employed as a supercapacitor. After Pb(II) adsorption, *C_p_* values were found to decrease, suggesting the increase in ion conductivity due to charge transfer (i.e., coordinative attachment)-type bonding of Pb(II) with **MXTP**. The variation in *C_p_* values of **MXTP** and Pb(II)-**MXTP** with the increasing scan rate is depicted in Figure 7c.

### 2.11. Conductance of MXTP and Pb(II)-MXTP

**Figure 8:**
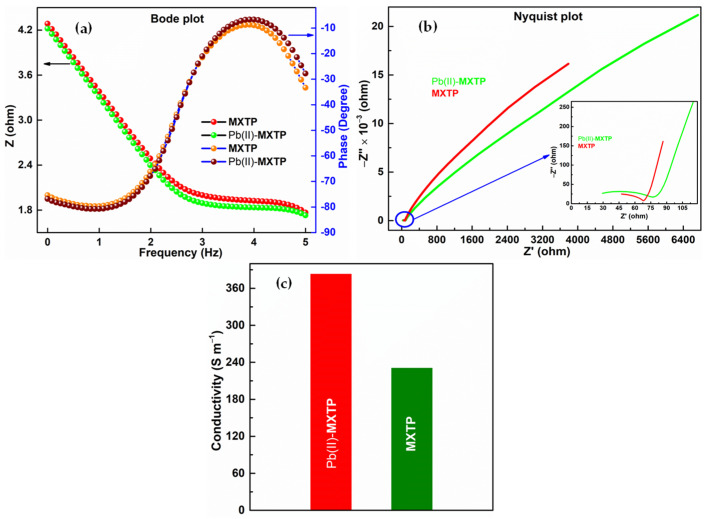
(**a**) Bode plot of **MXTP**/Pb(II)-**MXTP**, (**b**) Nyquist plot of **MXTP**/Pb(II)-**MXTP**, and (**c**) conductivities of **MXTP**/Pb(II)-**MXTP**.

In order to confirm the increase in conductivity of Pb(II)-**MXTP**, EIS experiments of **MXTP** and Pb(II)-**MXTP** were carried out within the frequency range of 0.1–100,000 Hz. Figure 8a shows the Bode plot of **MXTP** and Pb(II)-**MXTP**. From the Nyquist plot (Figure 8b), the initial point of the semicircle, known as solution resistance (*R_s_*, ohm), of **MXTP** and Pb(II)-**MXTP** were measured to be 49.37 and 28.76 ohm, respectively. By considering the area and length of GSE to be 7.10 × 10^−6^ m^2^ and 0.08 m, respectively, the conductivity values of **MXTP** and Pb(II)-**MXTP** were 228.23 and 391.78 S m^−1^, respectively (Figure 8c).

The CV and EIS experiments indicate that **MXTP** possesses very high specific capacitance at room temperature, whereas after Pb(II) adsorption, **MXTP** transforms into a conducting material. The ability of Pb(II)-**MXTP** to behave as a conducting polymer clearly indicates the successful post-adsorptive application of Pb(II) on the MXene-grafted hydrogel.

## 3. Conclusions

In this work, we have developed a scalable and reusable multifunctional MXene-grafted terpolymer hydrogel for the adsorptive application of Pb(II). The hydrogel is prepared by polymerizing hydrophilic monomers, such as AA and HEMA, in the presence of MXene in its aqueous phase. Optimization of the synthesis parameters has been carried out by synthesizing a series of hydrogels with strategically varied compositions and temperatures. The as-obtained hydrogel has been characterized in detail by ^1^H NMR, FTIR, XPS, XRD, TGA, DTG, and FESEM. The formation of MXene from the MAX phase, without using the conventional HF etching agent, has been confirmed from XRD, FTIR, and FESEM analyses. A strong coordinative association of MXene with **TP** is evident from XPS. Moreover, stronger coordinate bonding and weaker ion-exchange-type interactions of Pb(II) with –COO^−^/–O^−^ of **MXTP** have been indicated by FTIR, XPS and XRD analyses and manifested in the fitting of Langmuir and pseudo-second-order models. After adsorption, the Pb(II)-**MXTP** hydrogel has been tested for desorption and re-adsorption, which clearly points towards the recyclability of the adsorbent. In addition, the Pb(II)-**MXTP** hydrogel exhibits an attractive balance between specific capacitance and conductivity. There is a dearth of reports on the direct post-adsorptive application of Pb(II)-adsorbed material. To the best of our knowledge, the present work represents the first demonstration of such post-adsorptive application of Pb(II)-adsorbed material as a solid electrolyte/anode material. Enhancements in the scope of the use of such post-adsorptive materials could be sought based on further scrutiny of the polymer components and method of preparation. These efforts are currently underway in our laboratory.

## 4. Materials and Methods

### 4.1. Materials

MAX (Ti_3_AlC_2_), hydrochloric acid (HCl), calcium fluoride (CaF_2_), AA, HEMA, *N*,*N′*-methylenebisacrylamide (MBA), sodium hydroxide (NaOH), potassium persulfate (K_2_S_2_O_8_), sodium bisulfite (NaHSO_3_), and methanol were procured from Sigma (Maharashtra) and used directly. All experiments were conducted at 298 ± 2 K unless otherwise specified. Distilled water (DW) was used to perform experiments in the aqueous phase.

### 4.2. Synthesis of MXene-Grafted Terpolymer Hydrogel

#### 4.2.1. Synthesis of Mxene from MAX

We used the unconventional etching procedure of the MAX phase to avoid the involvement of high temperature and corrosive HF, the primary etching agent. Herein, HF was generated in situ by reacting CaF_2_ with concentrated HCl. In brief, 1 g dried MAX powder was taken in a double neck round bottom flask, followed by the addition of 5.2 g CaF_2_. The flask was heated to 333 K in a water bath. One neck of the flask was sealed with a N_2_ balloon and 30 mL conc. HCl was added dropwise through the other neck to remove the interlaying Al layers of MAX. After 30 min of etching, solid MXene was isolated by centrifugation, washed thoroughly by DW until acid-free, and finally dried in a vacuum oven.

#### 4.2.2. Synthesis of **TP** and **MXTP**

**Scheme 1:**
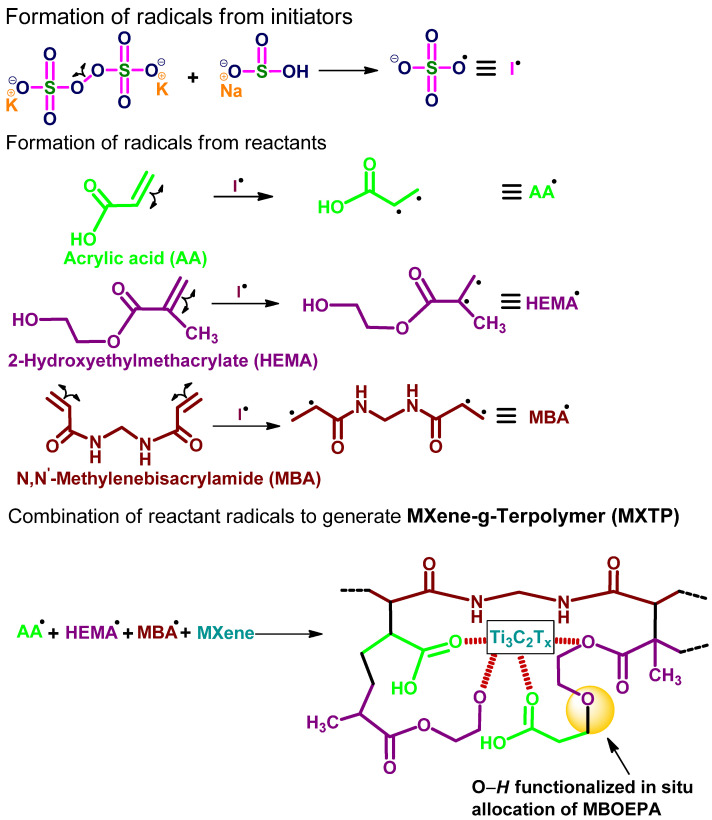
One-pot synthesis technique of **MXTP**.

The maximum adsorption capacity in relation to the structural integrity of hydrogel was ensured by optimizing the equilibrium swelling ratio (ESR, g/g) of a series of poly[AA-co-HEMA] hydrogels obtained by varying synthesis parameters. The parameters varied include AA/HEMA (–/*A*) within 5–20; amounts of MBA (wt.%/*B*), K_2_S_2_O_8_ + NaHSO_3_ (wt.%/*C*), and total monomers (wt.%/*D*) in the range of 1.0–2.0, 1.0–2.0, and 10–30 wt.%, respectively; and temperature (K/*E*) within the range of 288–308 K. Notably, despite having faster gelation beyond 308 K, such hydrogels have not been considered here to minimize the environmental impact of the use of synthetic constituents. The optimized conditions of synthesis were found to be 10, 1.5 wt.%, 1.5 wt.%, 20 wt.%, and 298 K for *A*, *B*, *C*, *D*, and *E*, respectively (Table S1). For the synthesis of **TP** (Scheme 1), 27.15 mL 8.66 (M) AA was neutralized to pH = 5.5 by NaOH, followed by the addition of 2.85 mL HEMA and 10 mL 0.97 (M) MBA solution. To this solution, 40 mL water was added and homogenized for 2 h on a magnetic stirrer. The homogeneous solution was purged with N_2_ gas and polymerization was initiated by the addition of redox initiators (10 mL of 0.28 M K_2_S_2_O_8_ and 10 mL of 0.72 M NaHSO_3_). Gelation was observed after 34 min and the yield was 81.4%. The as-obtained **TP** was collected on a Petri dish, washed with 1:3 (*v*/*v*) water/methanol solution, and dried in a hot air oven for 48 h. **MXTP** was synthesized by an analogous protocol by the addition of 0.5 g MXene to the mixture containing partially neutralized AA, HEMA, and MBA solutions. Subsequent polymerization and work-up were achieved as described above.

### 4.3. Characterization

Structural elucidation of **TP** was carried out by ^1^H NMR (Ascend NMR-500 MHz, Bruker; spectra acquired in DMSO-d_6_ solvent by 48 scans). Again, structures of **TP**, **MXTP**, and Pb(II)-**MXTP** were characterized by FTIR (Spectrum-2, PerkinElmer, Singapore; spectra acquired in solid state by using diamond plate and 16 scans), XPS (Omicron ESCA, Oxford Instrument Germany; spectra acquired in solid state and binding energy calibration was carrying out by taking adventitious carbon at 284.6 eV), TG (TGA 4000, PerkinElmer; thermal analysis was performed in N_2_-atmosphere within 20–850 °C and at the scanning rate of 10 °C min^−1^), XRD (D8 Discover, Bruker; data was acquired within 2θ = 5–75° at the scanning rate of 0.005° min^−1^), and FESEM (JSM7600F, Jeol, Tokyo, Japan), analyses. All chemical structures and graphical presentations were generated by ChemDraw Ultra 12.0 and Origin 9.0 software, respectively. Deconvolution of XPS plots was carried out by using the Gaussian function because of the higher adj. *R*^2^ and lowest *χ*^2^ values compared to other functions. Baseline corrections of XPS curves were carried out by using the Tougaard function.

### 4.4. Estimation of pH at Point of Zero Charge (pH_PZC_)

In total, 0.01 g dry MXTP was dipped into 50 mL buffered solutions of initial pH (pH_i_) ranging from 2 to 10. After dipping for 72 h, the pH of these buffer solutions was measured (pH_f_). The change in pH, i.e., ΔpH = pH_f_ − pH_i_, was plotted against pH_i_ to obtain pH_PZC_.

### 4.5. Adsorption Methodology

Isothermal adsorption experiments were carried out by inserting 0.02 g of oven-dried **MXTP** into 50 mL buffered solution of Pb(II) with 10–100 mg L^−1^ at pH 7. Adsorption isotherm and kinetics data were determined by measuring the unadsorbed Pb(II) concentrations (in mg L^−1^) at different time intervals via inductively coupled plasma mass spectrometry, i.e., ICPMS (Nexion 2000B ICP-MS, Perkin Elmer, Waltham, MA, USA). The equilibrium ACs (mg g^−1^), rate constants, and thermodynamics parameters were estimated using methods reported elsewhere [1,6,11].

### 4.6. Electrochemical Measurements

Specific capacitance and conductivity values of **MXTP**/Pb(II)-**MXTP** were estimated by CV and EIS analyses, respectively.

CV analysis. The CV experiments were carried out using a three-electrode module containing (a) a glassy silicon electrode (GSE)—the working electrode—(b) the reference electrode (RE)—Ag(s)/AgCl(aq) half-cell electrode—and (c) the counter electrode (CE)—a clean Pt wire applied for maintaining electrical neutrality of GSE. The working solution was prepared by dispersing 5 mg of samples in 5 and 40 μL of nafion and IPA, respectively, with the help of a vortex and sonicator. After drop-casting 5 μL of the as-prepared suspension on GSE, all three electrodes were dipped into 10 mL of buffer solution (pH = 7.0) and connected to a potentiostat. The current (*I*, *A*) in GSE can be expressed as [60,61,62]:

(1) $$I=C_{p}\times m\times k$$

Here, *C_p_* (F g^−1^), *m* (g), and *k* (V s^−1^) represent specific capacitance, mass of electroactive material (drop-casted on GSE), and scanning rate, respectively. From Equation (1), *C_p_* can be calculated by using Equation (2) (Text S1):

(2) $$C_{p}=\frac{A}{2(V_{2}-V_{1})mk}$$

EIS analysis. EIS experiments were carried out using a similar three-electrode module, containing GSE, RE, and CE. During EIS, alternating potential is sent towards the sample on GSE, and the corresponding alternating current (*i*, *A*) signal is recorded. From EIS, values of real impedance (*Z′*, ohm), imaginary impedance (*Z″*, ohm), vector sum of *Z′* and *Z″* (*Z_mag_*, ohm), amplitude (*ω*, Hz), and phase (*φ*, –) of an electroactive material can be obtained. From there, Bode and Nyquist plots are drawn, and from the solution resistance (*R_s_*, ohm) of Nyquist plot, conductivity (*σ*, ohm m^−1^) of the sample can be calculated (Equation (3) and Text S2) [63]:

(3) $$(\sigma )=\frac{1}{R_{S}}\times \frac{L}{A}$$

Here, *L* (m) and *A* (m^2^) represent the thickness and area of GSE, respectively.

## Figures and Tables

**Table 1:** FTIR analysis of MAX, MXene, **TP**, **MXTP**, and Pb(II)-**MXTP**.

Peaks (cm^−1^)					Explanation
MAX	MXene	TP	MXTP	Pb(II)-MXTP	
Absent	3455	3340/3193	3442–3108 (br)	3341/3185	Hydrogen-bonded O–H *str.* (originated from terminal O–H and –COOH in MXene and **TP**/**MXTP**/Pb(II)-**MXTP**, respectively [33,35,36,39]
Absent	2970	2947	2946	2941	C–H *str.* [10,11]
Absent	Absent	1703	1675	1685	Hydrogen-bonded >C=O *str*. of –COOH, shifting indicated the presence of coordinate bonding with Ti(IV) of MXene [1,36,40]
Absent	Absent	1554	1540	1535	>C=O *asym. str.* of –COO^−^ [10,11,19,36]
Absent	Absent	1396	1390	1396	–CH_2_– *def.* band of –CH_2_–CO–O– group of ester [36]
Absent	1350	Absent	1330/1037	1332	C–F band (formed in MXene due to F^−^ terminal group) [36,37]
Absent	Absent	1186	1182	1188	C–O–C *asym. str.* (Proof of O–C coupled grafting of third comonomer) [19,36]
Absent	Absent	1111	1081	1080	C–O–C *sym. str.* (Proof of O–C coupled grafting of third comonomer) [36]
Absent	528	Absent	525	525	Ti–O band (formed in MXene due to –OH terminal group) [37,38]
497/420	Absent	Absent	Absent	Absent	Al–C [34]
463	463	Absent	463	465	Ti–C [34]

**Table 2:** XPS analysis of MXene, **MXTP**, and Pb(II)-**MXTP.**

Orbitals	BEs (eV)			Assignment	Explanation
	MXene	MXTP	Pb(II)-MXTP		
C1s	NP *	283.99	283.83	Carbide from Mxene [43]	After Pb(II) adsorption, all the saturated carbon moieties appeared at an intermediate BE.
	NP	284.48	284.61	–**C**H_2_–/–**C**H< [40,44]	
	NP	285.60	285.91	**C**–O–**C** [11,20]	Shifting by +0.31 eV indicated the involvement of oxygen atom in coordinate bonding with Pb(II)
	NP	287.38	287.53	–**C**OOH/–**C**OO^−^[6,40,45,46]	Shifting by +0.15 eV indicated the involvement of oxygen atom in coordinate bonding with Pb(II)
O1s	NP	530.36	Absent	–C=(**O**)–OH [9]	Because of deprotonation at pH > pH_PZC_, the population of –COOH would be negligible
	NP	531.09	532.37	–C**OO**^−^ [11,45]	Shifting by +1.28 eV after Pb(II) adsorption indicated strong coordinate bonding of Pb(II) with –COO^−^ of **MXTP**
	NP	532.06/532.88	534.83	–**O**H termination group of Mxene [47]/–C=(O)–**O**H [11,48,49]	Since Pb(II) adsorption was carried out at pH > pH_PZC_, all –OH became deprotonated. Therefore, shifting by +2.77 and +1.95 eV inferred coordinate bonding of Pb(II) with –O^−^ of both polymer and MXene terminal –OH.
Ti2p_3/2_/ Ti2p_1/2_	453.65/459.69	450.91/455.93	NP	**Ti**–C–T_o_ [42]	–OH termination group, Strong coordinate bonding with –COO^−^ of **TP**
	454.91/460.95	451.89/458.08	NP	**Ti**–C–T_o,F_ [42]	F^−^ termination group
	457.76/463.48	–	NP	**Ti**–C–T_o_ [42]	–OH termination group
Pb4f	–	–	138.25/139.66	Pb4f_7/2_ [1,6]	Shifting by −1.05/+0.36 and −0.64/+0.55 eV from 139.30 and 143.80 eV for Pb 4f_7/2_ and Pb 4f_5/2_, respectively, of Pb(NO_3_)_2_ indicate stronger coordinate bonding/weaker ion-exchange-type interactions within –COO^−^ of **MXTP** and Pb(II)
	–	–	143.16/144.35	Pb4f_5/2_ [1,6]	

* not performed.

**Table 3:** Adsorption isotherm and kinetics parameters during Pb(II) adsorption onto **MXTP**.

Temperature (K)				
Model/Parameters	298	303	308	313
**Langmuir (adsorption isotherm parameters)**				
*q*_max_ (mg g^−1^)/pH_i_/*C*_0_ (mg L^−1^)	175.36/7/10–100	162.44/7/10–100	154.21/7/10–100	145.41/7/10–100
*k*_L_ (L mg^−1^)	0.2951	0.2145	0.1632	0.1688
*R*^2^/*F*	0.9981/4632.99	0.9976/4082.77	0.9937/1554.25	0.9996/23,505.24
*χ* ^2^	8.1245	7.8969	18.1518	1.1116
**Pseudo-second-order (adsorption kinetics parameters)**				
*q*_e,cal_ (mg g^−1^)/pH_i_/*C*_0_ (mg L^−1^)	166.69/7/100	151.69/7/100	137.04/7/100	131.29/7/100
*q*_e,exp_ (mg g^−1^)	160.46 ± 4.82	148.44 ± 4.31	138.61 ± 4.29	129.56 ± 4.16
*k*_2_ (g mg^−1^ min^−1^)	3.81 × 10^−4^	6.18 × 10^−4^	10.50 × 10^−4^	13.31 × 10^−4^
*R*^2^/*F*	0.9883/5501.01	0.9841/4329.76	0.9829/3258.39	0.9981/55,434.29
*χ* ^2^	2.5779	2.8968	3.2276	1.6262

**Table 4:** Adsorption thermodynamics parameters during Pb(II) adsorption onto **MXTP**.

Concentration (ppm)/Temperature (K)	−Δ*G*^0^ (kJ mol^−1^)	−Δ*H*^0^ (kJ mol^−1^)	−Δ*S*^0^ (J mol^−1^ K^−1^)
5/298	9.68	47.07	125.46
5/303	9.05		
5/308	8.43		
5/313	7.79		
10/298	8.72	36.51	93.45
10/303	8.22		
10/308	7.77		
10/313	7.3		
15/298	7.82	45.38	126.04
15/303	7.19		
15/308	6.56		
15/313	5.93		
20/298	6.39	43.86	125.79
20/303	5.77		
20/308	5.14		
20/313	4.51		
25/298	4.84	32.05	91.29
25/303	4.39		
25/308	3.93		
25/313	3.48		
30/298	3.69	26.26	75.74
30/303	3.32		
30/308	2.94		
30/313	2.56		

**Table 5:** Comparative behavior of reported adsorbents towards Pb(II).

Adsorbate	Name of Adsorbents	Adsorption Capacities (mg g^−1^) /pH_o_/*C*_0_ (ppm)/Temperatures (K)	Reference
Pb(II)	IPNS ^a^	54.86/7.00/5–30/303	[1]
	Bare malachite nanoparticle	7.20/5.0–6.0/10–100/–	[53]
	Kaolinite	11.50/5.7/10–50/303	[54]
	Montmorillonite	31.10/5.7/10–50/303	[54]
	Jordanian kaolinite	13.32/5.0/50–400/295	[55]
	GGAAAMAPA ^b^	41.98/7.00/5–25/303	[40]
	ICZ ^c^	2.28/6.5/50/298	[56]
	APAN ^d^	60.60/4.0/40–1000/303	[57]
	Fe_3_O_4_^e^	22.83/5 ± 0.2/20/303	[58]
	Lemon peel	37.87/5.0/100–300/301	[59]
	**MXTP ^f^**	**175.36/7.00/10–100/298**	**PS ^^^**

^a^ pectin-g-(sodium acrylic acid-co-*N*-isopropylacrylamide), ^b^ guar gum-g-(acrylic acid-co-acrylamide-co-3-acrylamido propanoic acid), ^c^ iron-coated zeolite, ^d^ aminated polyacrylonitrile, ^e^ iron oxide, ^f^ MXene-grafted poly[acrylic acid-co-2-hydroxyethyl methacrylate], and ^^^ present study.

## Data Availability

We would like to share data of this article in ResearchGate.

## Supplementary Materials

The following supporting information can be downloaded at: https://www.mdpi.com/article/10.3390/gels9100827/s1, Figure S1: FTIR of MAX and MXene; Figure S2: pH_PZC_ of **MXTP**; Figure S3: XRD of MAX and MXene; Figure S4: Wide-scan survey plot of **TP** and **MXTP**; Table S1: Optimization of synthesis parameters for **TP** and **MXTP**; Text S1: Derivation of working formula for CV analyses; Text S2: EIS analyses.
