# Supplementary material for: An MXene-Grafted Terpolymer Hydrogel for Adsorptive Immobilization of Toxic Pb(II) and Post-Adsorption Application of Metal Ion Hydrogel
Source: Gels. 2023 Oct 19;9(10):827. doi: 10.3390/gels9100827 (PMC10606399; doi:10.3390/gels9100827)
Supplement: Supplementary file 1 [file gels-09-00827-s001.zip › gels-2663445-supplementary.pdf]

A MXene-grafted-terpolymer hydrogel for adsorptive immobilization of toxic Pb(II) and post-adsorption application of metal ion hydrogel

Himarati Mondal[*,a], Mrinmoy Karmakar[*,b,c] and Bhaskar Datta[*,a,d]

[a]Department of Chemistry, Indian Institute of Technology Gandhinagar, Palaj, Gujarat-382055, India
[b]Department of Chemical Engineering, Indian Institute of Technology Gandhinagar, Palaj, Gujarat-382055, India
[c]Presently in Department of Pharmacy, College of Pharmacy, Kangwon National University, Chuncheon, Gangwon 24341, Republic of Korea
[d]Department of Biological Engineering, Indian Institute of Technology Gandhinagar, Palaj, Gujarat-382055, India
*Correspondence: authors: himarati.m@iitgn.ac.in/ himaratimondal236@gmail.com (H.M.); mrinmoy.enterprise@gmail.com (M.K.); and bdatta@iitgn.ac.in (B.D.)

Index

Entries	Details	Pages
Table S1	Optimization of synthesis parameters for **TP** and **MXTP**	S3
Text S1	Derivation of working formula for CV analyses.	S4
Text S2	EIS analyses	S4
Figure S1	FTIR of MAX and MXene	S5
Figure S2	pH_{PZC} of **MXTP**	S6
Figure S3	XRD of MAX and MXene	S6
Figure S4	Wide-scan survey plot of **TP** and **MXTP**	S7

Table S1. Optimization of synthesis parameters for **MXTP.**

Hydrogel	*A* (–)	*B* (wt.%)	*C* (wt.%)	*D* (wt.%)	*E* (K)	MXene (wt.%)	ESR(s) (g/g)
A	5.0	1.5	1.0	20.0	298	0.50	418.50 ± 12.56
**B**	**10.0**	**1.5**	**1.0**	**20.0**	**298**	**0.50**	**558.00 ± 16.74**
C	20.0	1.5	1.0	20.0	298	0.50	502.20 ± 15.07
D	10.0	1.0	1.0	20.0	298	0.50	491.04 ± 14.71
**B**	**10.0**	**1.5**	**1.0**	**20.0**	**298**	**0.50**	**558.00 ± 16.74**
E	10.0	2.0	1.0	20.0	298	0.50	513.36 ± 15.41
B	10.0	1.5	1.0	20.0	298	0.50	558.00 ± 16.74
**F**	**10.0**	**1.5**	**1.5**	**20.0**	**298**	**0.50**	**620.00 ± 18.60**
G	10.0	1.5	2.0	20.0	298	0.50	570.40 ± 17.11
H	10.0	1.5	1.5	10.0	298	0.50	551.80 ± 16.55
**F**	**10.0**	**1.5**	**1.5**	**20.0**	**298**	**0.50**	**620.00 ± 18.60**
I	10.0	1.5	1.5	30.0	298	0.50	576.60 ± 17.29
–	10.0	1.5	1.5	20.0	288	0.50	No gelation
**F**	**10.0**	**1.5**	**1.5**	**20.0**	**298**	**0.50**	**620.00 ± 18.60**
J	10.0	1.5	1.5	20.0	308	0.50	589.00 ± 17.67
K	10.0	1.5	1.5	20.0	288	0.25	415.40 ± 12.46
**F**	**10.0**	**1.5**	**1.5**	**20.0**	**298**	**0.50**	**620.00 ± 18.60**
L	10.0	1.5	1.5	20.0	308	0.75	520.80 ± 15.62
**F (optimum)**	**10.0**	**1.5**	**1.5**	**20.0**	**298**	**0.50**	**620.00 ± 18.60**

Text S1. Derivation of working formula for CV analyses. The current (I, A) in GSE can be expressed as:

$$\mathrm{I} = C_p \times m \times k \tag{S1}$$

Here, C_p (F g^{-1}), m (g), and k (V s^{-1}) represent specific capacitance, mass of electroactive material (drop-casted on GSE), and scanning rate, respectively.
When potential of GSE was varied within V_1–V_2 (V), then Eq. (S1) can be reframed as:

$$\int_{V_1}^{V_2} \mathrm{I}(V)dV = \int_{V_1}^{V_2} (C_p \times m \times k)dV \tag{S2}$$

Since, $\int_{V_1}^{V_2} \mathrm{I}(V)dV$ can be related to area under CV curve (A, A V), Eq. (S2) modifies as:

$$A = C_p mk \int_{V_1}^{V_2} dv \tag{S3}$$

$$\text{Or, } A = C_p mk(V_2 - V_1) \tag{S4}$$

In charging condition of a capacitor, A is replaced by A_1 and Eq. (S4) transforms to:

$$A_1 = C_p mk(V_2 - V_1) \tag{S4A}$$

However, in discharging condition, A is replaced by A_2 and Eq. (S4) is modified to:

$$A_2 = C_p mk(V_1 - V_2) \tag{S4B}$$

Therefore, area under CV curve, i.e., A can be calculated as:

$$A = A_1 - A_2 = 2[C_p mk(V_2 - V_1)] \tag{S5}$$

Eq. (S5) can be rearranged in terms of C_p:

$$\mathrm{C}_p = \frac{A}{2(V_2 - V_1)mk} \tag{S6}$$

C_p values of **MXTP** and Pb(II)-**MXTP** were estimated by using Eq. (S6).

Text S2. *EIS analyses.* EIS experiments were carried out using a three-electrode module, composed of GSE, RE, and CE. During EIS, alternating potential is sent towards the sample on GSE, and the corresponding alternating current (i, A) signal is recorded. Therefore, input potential and output current can be written as shown in Eq. (S7) and Eq. (S8), respectively:

$$E(t) = E_o \sin(\omega t) \tag{S7}$$

$$i(t) = i_o \sin(\omega t) \tag{S8}$$

Here, t (s), i_0 (A)/ E_0 (V), and ω (Hz) represent time, amplitude of current/ potential, and angle of frequency, respectively.
However, phase shift (φ, degree) is observed for output current. Hence, Eq. (S8) can be modified as:

$$i(t) = i_o \sin(\omega t - \varphi) \tag{S8A}$$

For AC output system, impedance (Z, ohm) can be defined as:

$$Z = \frac{E}{i} \tag{S9}$$

The x and y components of Z, i.e., Z_x (ohm) and Z_y (ohm), respectively, can be evaluated as:

$$Z_x = Z_{mag} \cos\theta \tag{S10A}$$

$$Z_y = Z_{mag} \sin\theta \tag{S10B}$$

Here, Z_{mag} (ohm) is the vector sum of Z_x and Z_y and can be defined as:

$$Z_{mag} = \sqrt{Z_x^{\,2} + Z_y^{\,2}} = \frac{E_0}{i_0} \tag{S11}$$

Z_x and Z_y are also termed as real impedance (Z', ohm) whereas Z_z (Z'', ohm) is considered as imaginary impedance.

From EIS, values of Z_{mag}, Z', Z'', ω, and φ of an electroactive material can be obtained. From there, Bode and Nyquist plots are drawn, and from the solution resistance (R_s, ohm) of Nyquist plot, conductivity (σ, ohm m^{-1}) of the sample can be calculated by using the following equation:

$$(\sigma) = \frac{1}{R_S} \times \frac{L}{A} \tag{S12}$$

Here, L (m) and A (m^2) represent thickness and area of GSE, respectively.

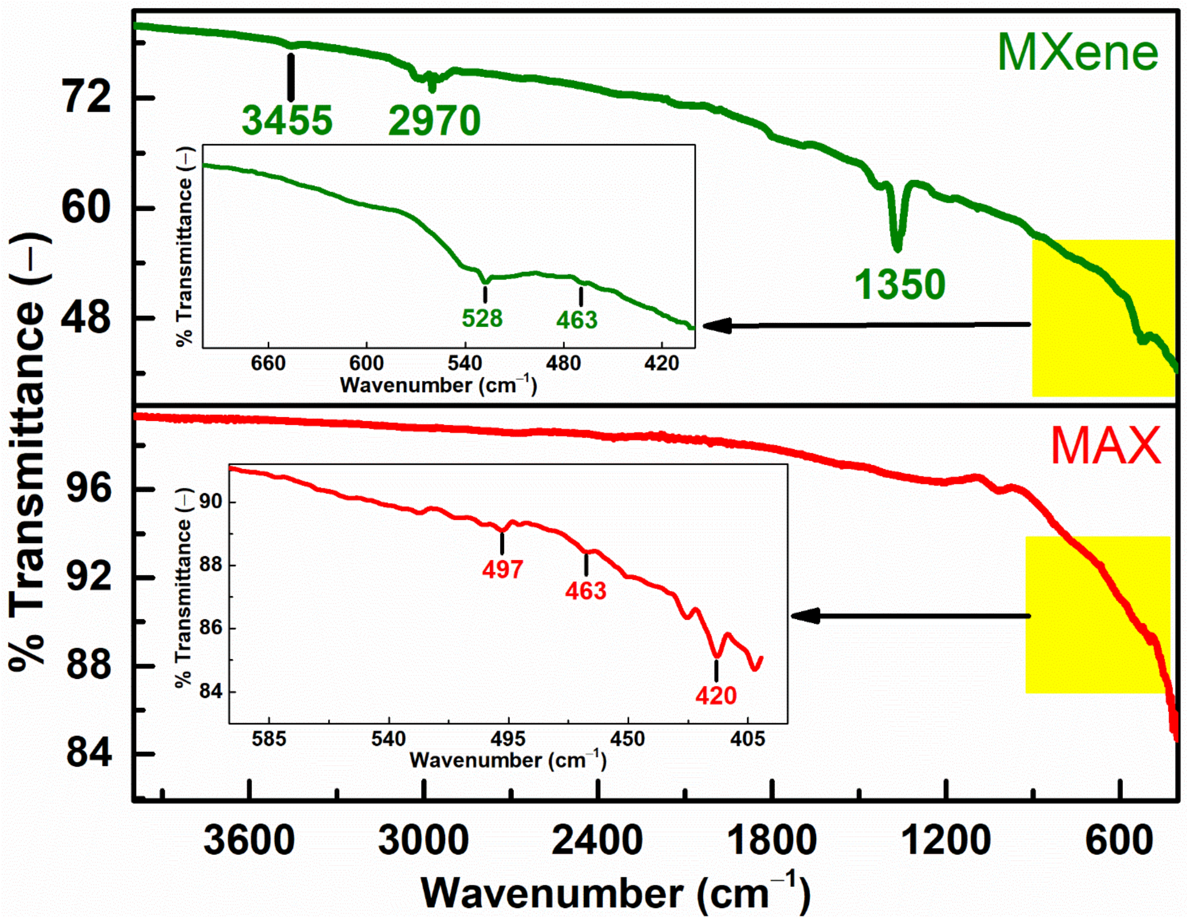

Figure S1. FTIR of MAX and MXene

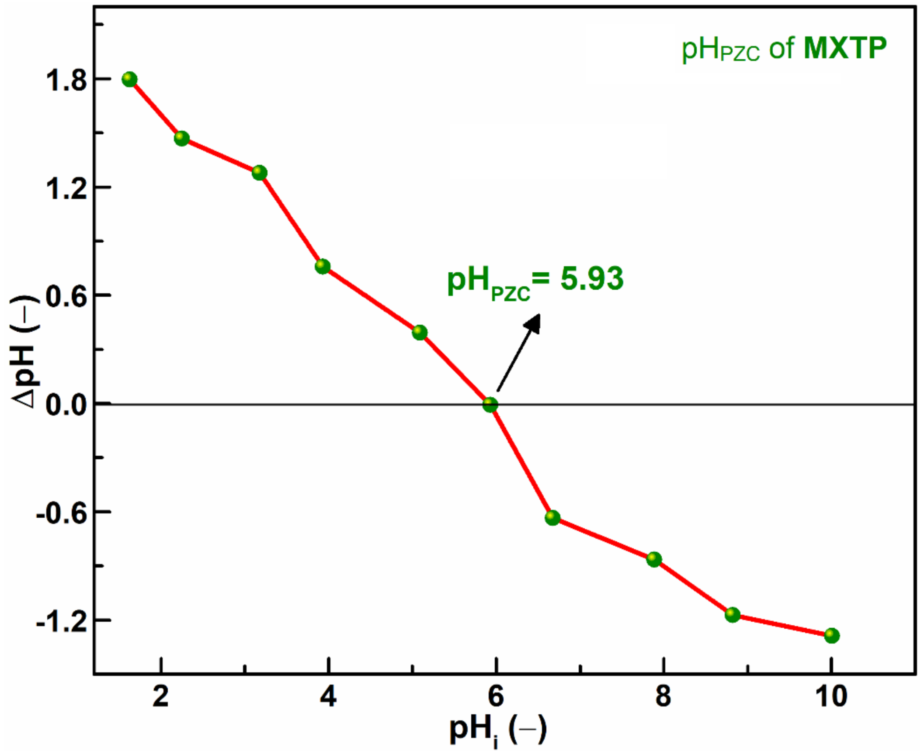

Figure S2. pHPZC of **MXTP**

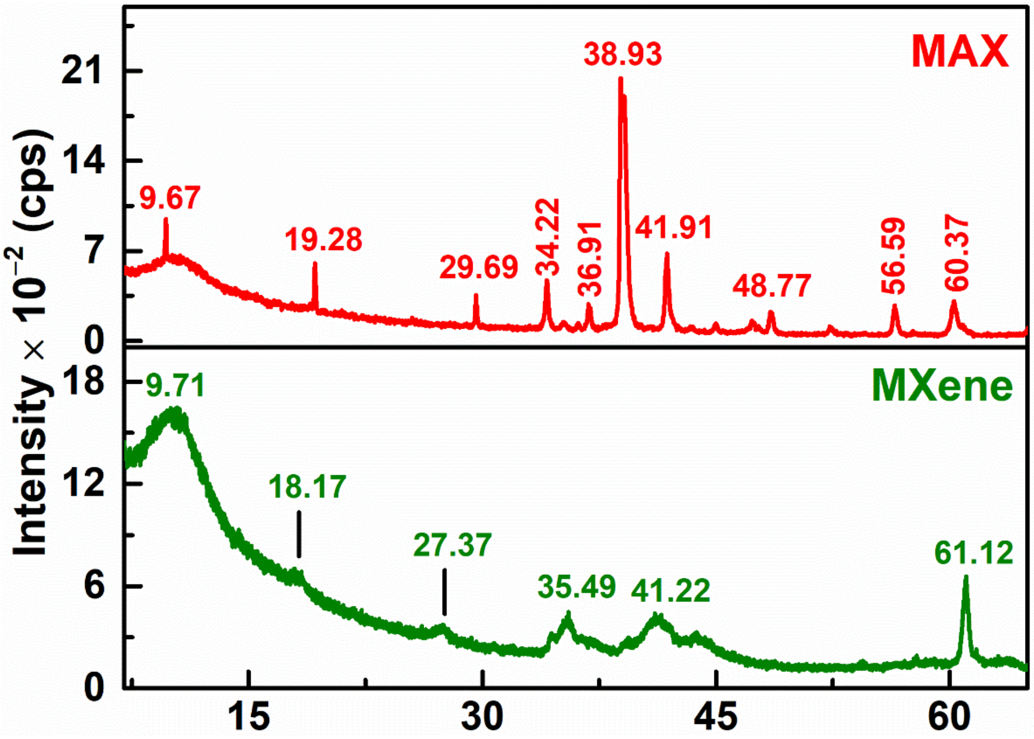

Figure S3. XRD of MAX and MXene

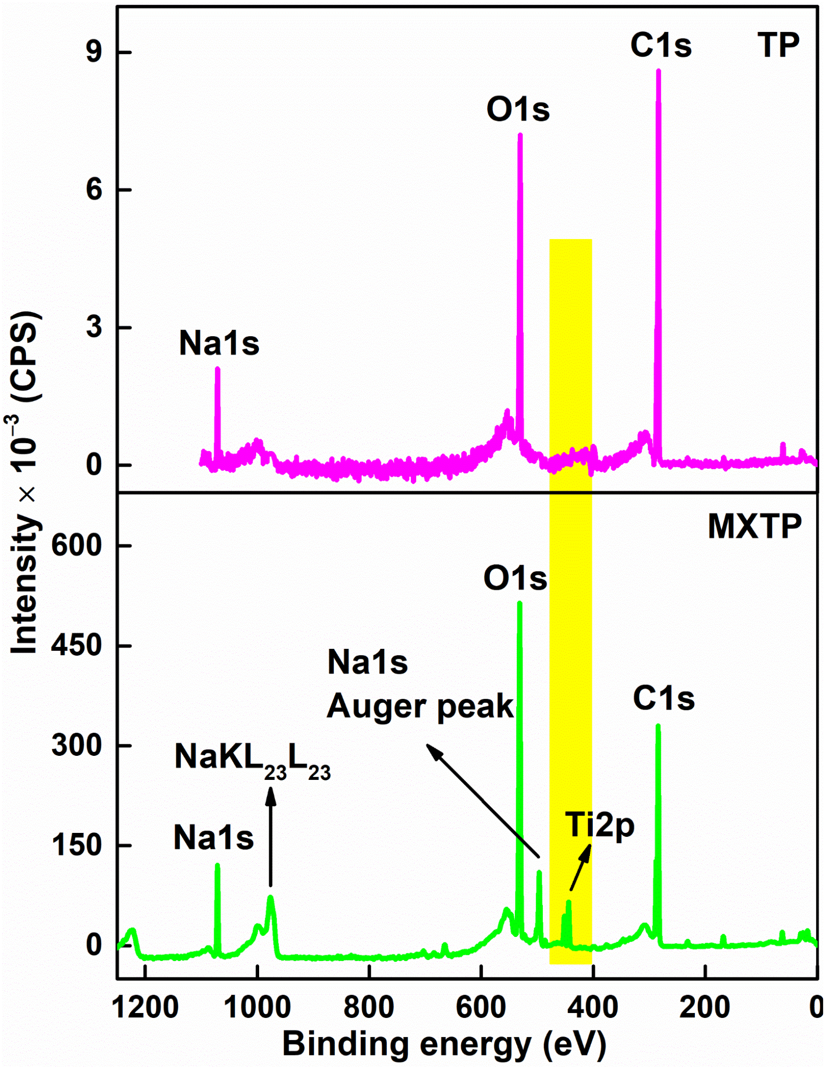

Figure S4. Wide-scan survey plot of **TP** and **MXTP**
